# A Case Series of Myocarditis Related to the COVID-19 Vaccine

**DOI:** 10.7759/cureus.29892

**Published:** 2022-10-04

**Authors:** Hayfa O Ahmed, Mawada M Ahmed, Omer Elrasheid

**Affiliations:** 1 Cardiology, Security Forces Hospital, Riyadh, SAU

**Keywords:** perimyocarditis, pericarditis, myocarditis, vaccine, covid-19

## Abstract

Perimyocarditis related to the coronavirus disease 2019 (COVID‐19) vaccine is one of the rare adverse events that emerged in April 2021 and then the number of cases commensurably increased as the number of vaccinated people rose. This is a case series of myocarditis/pericarditis related to the messenger RNA (mRNA) COVID‐19 vaccine in which we identified four cases with different presentations and outcomes. A short-term follow-up period of five months revealed a full recovery of three cases within one to 12 weeks and persistent left ventricular systolic dysfunction in the fourth case which will require further follow-up to assess long-term outcomes.

## Introduction

Myocarditis is one of the rare conditions among the general population and from previous vaccinations in the pre-coronavirus disease 2019 (COVID-19) era. The incidence increased with the COVID-19 infection and related vaccinations according to the Centers for Disease Control and Prevention (CDC) reports from October 6, 2021; a total of 402,469,096 doses of COVID-19 vaccines were administered from which there were 3,336 reports of myocarditis and pericarditis [1].

Myocarditis has multiple presentations with varying degrees of severity ranging from asymptomatic or subclinical disease with mild dyspnea and chest pain to sudden death due to cardiogenic shock or malignant ventricular arrhythmias [2,3].

Regarding the COVID-19 vaccine, the results of some trials revealed that the COVID-19 vaccine was effective, safe, and similar to those other vaccines [4].

## Case presentation

Case 1

A previously healthy 14-year-old male presented with mild chest discomfort which increased when lying flat, and it started on the fourth day following the second dose of messenger RNA (mRNA) COVID‐19 vaccine. He had a fever on the second day that responded to regular paracetamol and disappeared on the third day, however, no other symptoms were observed.

**Figure 1:**
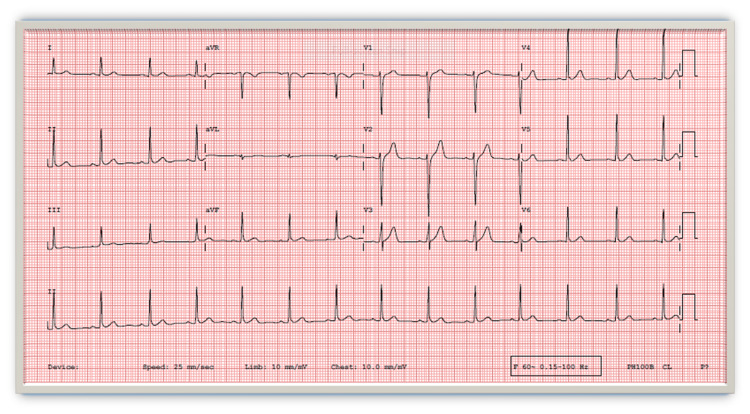
Normal ECG ECG: electrocardiogram

**Figure 2:**
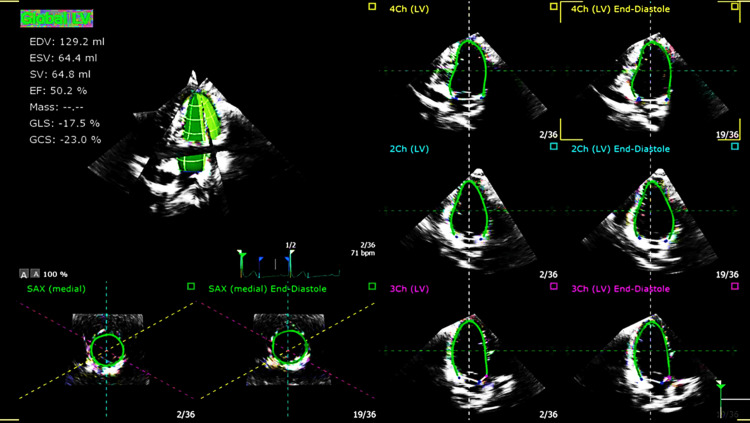
Transthoracic echo echocardiography showed the 3D full volume LVEF measurement which revealed an EF of 50%. 3D; 3 dimensional, LVEF; left ventricular ejection fraction, EF: ejection fraction.

**Figure 3:**
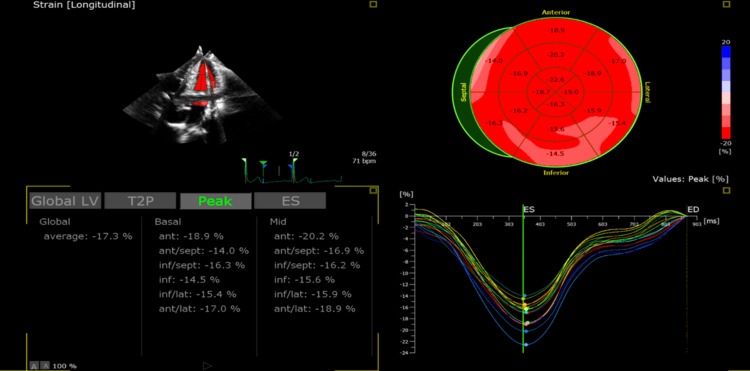
Transthoracic echo echocardiography showed a reduced GLS of 17% with variable regionality. GLS; global longitudinal strain.

It is worth mentioning that there was no evidence of COVID-19 infection, and the first dose of the COVID-19 vaccine was administered 24 days before the second dose which passed uneventfully. Cardiovascular and systemic examinations were unremarkable. Investigations showed a normal chest x‐ray and normal electrocardiogram (ECG) (Figure 1), elevated troponin and C-reactive protein (CRP) levels (Table 1), along with low normal left ventricle systolic function LVEF 50% by a three-dimensional transthoracic echocardiogram (3D TTE) (Figure 2) and global longitudinal strain (GLS) of -17% on transthoracic echocardiogram (TTE) (Figure 3). Based on the above, he was diagnosed with clinically suspected myocarditis related to the mRNA COVID-19 vaccine with mild left ventricular systolic dysfunction. The patient was admitted to the coronary care unit (CCU) for observation and treatment of myocarditis. Non-steroidal anti-inflammatory drugs (NSAIDs) and colchicine were initiated because of the pericarditic nature of the chest pain.

**Table 1:** Clinical and echocardiographic characteristics of four patients who presented with perimyocarditis related to the COVID-19 vaccine NA: not applicable proBNP: pro-brain natriuretic peptide; WBC: white blood cell count; ESR: erythrocyte sedimentation rate; CRP: C-reactive protein; LVEF: left ventricular ejection fraction; EF: ejection fraction

Data	Case 1	Case 2	Case 3	Case 4	Normal level
Age in years	14	22	16	33	NA
Sex	Male	Male	Male	Male	NA
History of cardiac disease	No	No	No	No	NA
Type of vaccine	Pfizer	Pfizer	Moderna	Pfizer	NA
Time from vaccination to presentations (days)	4	24	4	4	NA
COVID-19 vaccine dose	2^nd^ dose	1^st^ dose	2^nd^ dose	2^nd^ dose	NA
COVID-19 PCR	Negative	negative	Negative	Negative	Negative
Troponin level ng/ml	0.714	0.05	0.081	0.716	<0.014
proBNP pmol/l	5.58	NA	8.74	30	<53
WBCs (10*9/L)	10.41	6.2	14	10	4.5-13.5
ESR mm/hr	17	118	28	92	0-20
CRP mg/l	7.44	226	11.53	115	<5
Creatinine umol	72	129	82	79	60-110
LVEF (%) at presentation	50%	48%	54%	45%	52-70%
Medications	Ibuprofen and colchicine	Aspirin, colchicine and lisinopril	Aspirin and colchicine	Ibuprofen and colchicine	NA
Duration of hospitalization (days)	4	2	5	4	NA
Follow-up EF (%)	65%	45%	55%	60%	52-70%

He was discharged after four days when his chest pain resolved and the results of his blood tests including the troponin level improved (0.23). He was advised to avoid high-intensity and competitive sports for the next three to six months and a follow-up appointment was arranged. One week later, he was reassessed in the follow-up clinic, he had no symptoms and his TTE showed a normal left ventricular systolic function (LVEF) of 65%.

Final Diagnosis: A fully recovered clinically suspected myocarditis related to mRNA COVID-19 vaccine.

Case 2

**Figure 4:**
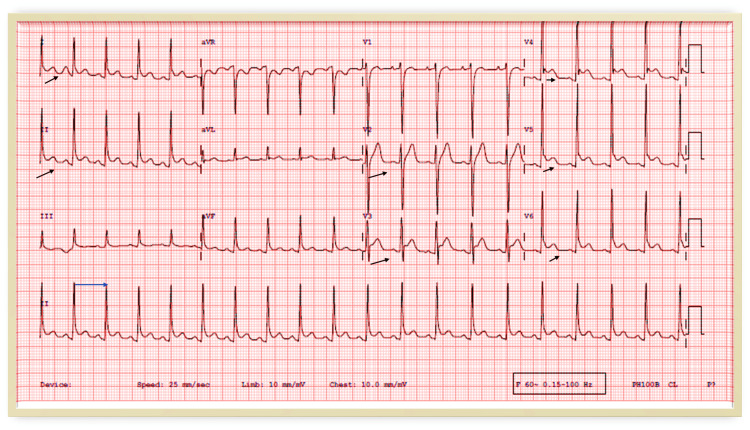
There is sinus tachycardia (blue arrow) and diffuse ST-segment elevation and PR depression consistent with pericarditis (black arrows).

A 22-year-old male who is a known case of bronchial asthma presented 24 days following the first dose of the mRNA COVID-19 vaccine with fatigability, then one week later he developed stabbing chest pain and low-grade fever. Clinical examination was unremarkable apart from a temperature of 37.6. His initial investigations showed no evidence of COVID-19 infection (negative COVID 19 PCR test), normal troponin level, elevated inflammatory markers (Table 1), sinus tachycardia and diffused ST elevation in the ECG, and PR depression (Figure 4). There was mild pericardial effusion on the TTE with normal LV systolic function. During the hospital stay, his condition deteriorated, and laboratory values showed high creatinine and elevated troponin levels. A chest X-ray and CT chest were normal; the TTE showed normal size left ventricle, mild global LV systolic dysfunction, and EF 48% with no significant valvular dysfunction.

The patient was diagnosed with perimyocarditis related to the COVID-19 vaccine and was observed in the hospital for seven days. Medication including aspirin and colchicine were started then discharged in good condition. On follow up the ECG changes have resolved but his LV ejection fraction remains at 45% until five months after the event.

The final diagnosis: A persistent left ventricular dysfunction following perimyocarditis related to the COVID-19 vaccine.

Case 3

**Figure 5:**
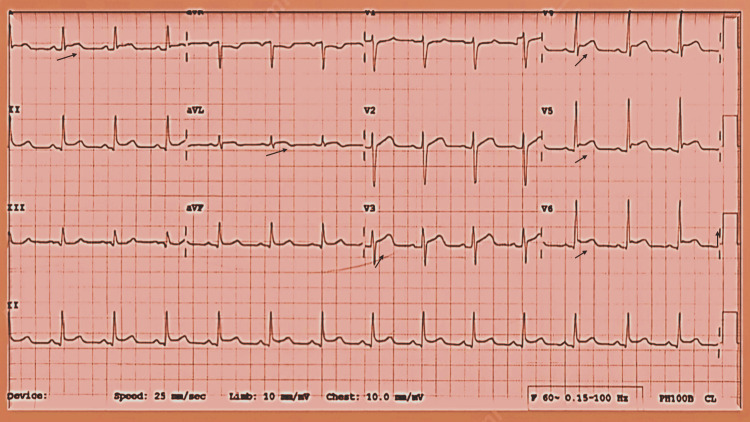
There is diffuse ST-segment elevation in the anterolateral leads (black arrows).

Case 3 describes a 16 years old male without underlying comorbidities who developed a fever on Day 4 following the second dose of mRNA COVID-19 vaccin lasting for one day and then resolving thereafter. Subsequently, on Day 13 from the vaccination, he developed a sore throat and substernal chest pain which increased with breathing and walking and subsided at rest, for which he presented to the emergency room. Systemic examination was unremarkable apart from tachycardia. The requested blood tests showed leukocytosis and elevated troponin, serum creatinine, and inflammatory markers (Table 1). His chest X-ray and TTE were normal, and ECG showed sinus tachycardia. He was admitted for observation and bed rest, and started on aspirin and colchicine. On the second day of admission, the ECG showed ST-segment elevation in the anterolateral leads (Figure 5), and TTE revealed a borderline reduced LV systolic function (EF 54%) with mild hypokinesia of the anterior septum and troponin level reached 1.2 ng/ml. Upon symptom resolution and normalization of troponin, the patient was discharged home. The follow-up TTE after two weeks showed a normal size left ventricle, normal global systolic LV function (EF 58%), and no regional wall motion abnormality.

Final diagnosis: Fully recovered patient with clinically suspected myocarditis related to mRNA COVID-19 vaccine.

Case 4

**Figure 6:**
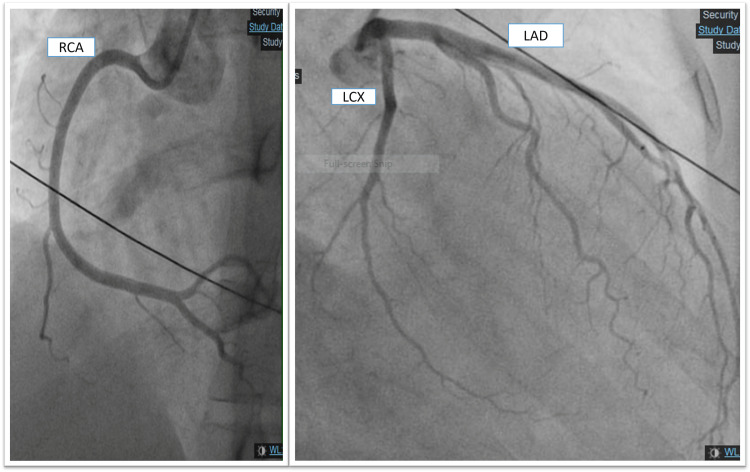
Coronary angiogram which shows non-obstructive coronary arteries. LAD; left anterior descending, LCX; left circumflex, RCA: right coronary artery.

**Figure 7:**
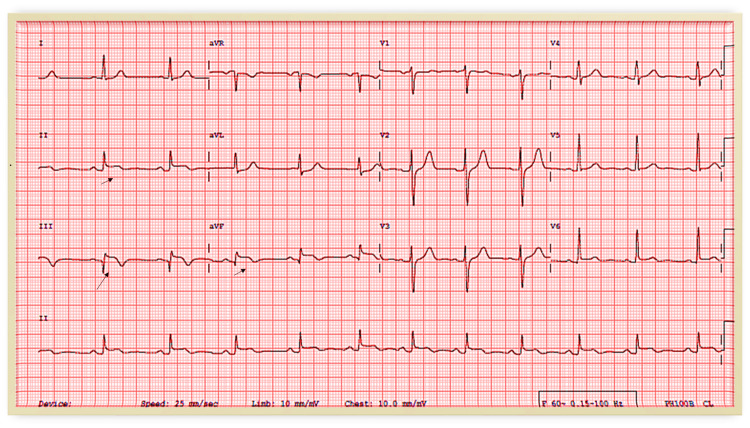
ECG showing ST-segment elevation in the inferior leads (black arrows) and q wave in lead III ECG: electrocardiogram

A previously healthy 33 year-old-male had a progressive dry cough which started on the fourth day following the second dose of mRNA COVID-19 vaccine; subsequently, he developed shortness of breath, chest pain, and palpitations which increased in severity over the following three weeks, after which he was admitted to the hospital. His clinical examination was unremarkable. Investigations showed elevated troponin, CRP, and ESR levels (Table 1), ECG showed ST-elevation in the inferior leads and q wave in lead III (Figure 6), TTE showed mildly reduced LV systolic function, EF 45%, and hypokinesia of the basal and mid inferior and inferolateral walls. Therefore, he was initially diagnosed with ST-elevation myocardial infarction, so coronary angiography was done and showed non-obstructed coronary vessels (Figure 7). Then he was diagnosed with myocarditis and observed, started on ibuprofen and colchicine, and discharged after two days when his condition and troponin level improved (0.40), and advised to avoid high-intensity and competitive sports for the next three to six months. During follow-up, valsartan and bisoprolol were added for high blood pressure and palpitations. Repeated TTE after two weeks showed improved LV systolic function with LVEF of 50% and no regional wall motion abnormality and by the third month, the LV function has normalized with LVEF of 60%.

## Discussion

The link between the COVID-19 vaccine and myocarditis/pericarditis is still unclear and poorly understood as the gold standard for the diagnosis is the histological or immunohistological evidence from a myocardial biopsy which is not done in most cases. However, multiple theories were raised previously about hypersensitivity and drug-induced myocarditis/pericarditis. One of the theories suggests that the immune system may detect the mRNA in the vaccine as an antigen, resulting in the activation of pro-inflammatory cascades and immunologic pathways that may play a role in the development of myocarditis as part of a systemic reaction in certain individuals [5]. 

Various cases of COVID-19 vaccine myocarditis have been described in different published reports [6-8]. As stated in one of these reports the majority were healthy young males without a history of COVID-19 infection, they presented two to three days after the second dose of mRNA COVID-19 vaccines with chest pain usually occurring with or without fever and myalgia one day after vaccination. All had an elevated cardiac troponin (the highest-level peaking usually three days after vaccination), additionally; B-type natriuretic peptide or N-terminal pro-B-type natriuretic peptide levels (pro-BNP) were only mildly elevated in approximately two-thirds of the patients when measured and C-reactive protein levels were elevated in most cases and decreased along with troponin through the hospital stay. ECG was abnormal with ST elevations in most presentations. Noninvasive imaging investigations such as the echocardiogram was abnormal in 40% (a small percentage had a left ventricular ejection fraction <50%), and cardiac MRI was abnormal in all tested patients (findings suggestive of myocarditis such as late gadolinium enhancement and myocardial edema were observed). Nearly all the patients had resolution of symptoms and signs and improvement in diagnostic markers and imaging with or without treatment [8].

This case series consists of four cases of suspected myocarditis/pericarditis related to the COVID-19 vaccine which was diagnosed according to the 2021 scientific statement from the American College of Cardiology/American Heart Association (ACC/AHA) as cardiac MRI is not available in our hospital [9,10].

In this case series, we observed that myocarditis/pericarditis related to the mRNA COVID‐19 vaccine occurs in young healthy males between 14-33 years following the second dose of Pfizer vaccine in most cases within four days to four weeks, especially on the fourth day of vaccination (Table 1). Variable presentations were noted such as fatigue, mild chest discomfort to severe chest pain, shortness of breath, palpitations, fever, and sweating. All patients had no evidence of COVID-19 infection and had an elevated troponin level and inflammatory markers, ECG changes in most patients consist of myocarditis /pericarditis such as sinus tachycardia, ST-elevation, and PR depression. Echocardiography showed variable degrees of LV systolic dysfunction but all in the mild to moderate range, and one patient had regional wall motion abnormality. The average duration of hospital admission was between two to seven days during which their symptoms and biochemical markers improved. The treatment strategies advised avoiding high-intensity and competitive sports for the next three to six months for all patients [11]. Those with evidence of pericarditis received NSAIDs and colchicine, bisoprolol and valsartan were added for palpitation and high blood pressure, respectively for one patient. Those with LV dysfunction and regional wall motion abnormality showed complete recovery in a period ranging from two weeks to three months except for one patient who had a persistent LV dysfunction beyond the fifth month.

Based on the above, our findings are in keeping with the published data regarding myocarditis and pericarditis related to the COVID‐19 vaccine focusing particularly on the persistent LV dysfunction that occurred as an adverse event following COVID-19 vaccination which will need further follow-up to determine long-term outcomes.

## Conclusions

We describe a case series of myocarditis and perimyocarditis related to the mRNA COVID‐19 vaccine with variable presentations and recovery. The unique finding of our study is the possibility of the long-term effect of vaccine-related myocarditis (this study demonstrates the short-term follow-up and picked up one case with persistent LV systolic dysfunction that has not fully recovered after five months). Additionally, we describe that myocarditis can occur following the first dose of vaccination in association with pericarditis.

Further surveillance and evaluation of this rare adverse event following vaccination is needed. This event can be included in the context of the well-established risk of morbidity and follow-up of long-term outcomes should be implemented. Despite these rare complications of the COVID-19 vaccination, it is still the cornerstone of prevention and its benefits outweigh the risks in decreasing the rate of infection, hospitalization, and serious complications.
